# Novel Tools for Prostate Cancer Prognosis, Diagnosis, and Follow-Up

**DOI:** 10.1155/2014/890697

**Published:** 2014-05-04

**Authors:** Andreas Dimakakos, Athanasios Armakolas, Michael Koutsilieris

**Affiliations:** Physiology Laboratory, Medical School, National and Kapodistrian University of Athens, Mikras Asias 75, 11527 Athens, Greece

## Abstract

Prostate-specific antigen (PSA) is the main diagnostic tool when it comes to prostate cancer but it possesses serious limitations. Therefore, there is an urgent need for more sensitive and specific biomarkers for prostate cancer prognosis and patient follow-up. Recent advances led to the discovery of many novel diagnostic/prognostic techniques and provided us with many worthwhile candidates. This paper briefly reviews the most promising biomarkers with respect to their implementation in screening, early detection, diagnostic confirmation, prognosis, and prediction of therapeutic response or monitoring disease and recurrence; and their use as possible therapeutic targets. This review also examines the possible future directions in the field of prostate cancer marker research.

## 1. Introduction

Prostate cancer is the sixth leading cause of cancer-related death in men (it is now the second in the United States and first in the UK) [1]. While there are exceptions, it is not a particularly aggressive form of cancer, and it tends to metastasize mainly to bones and lymph nodes [2]. Many factors have been proven to be implicated in the development of prostate cancer, including diet and genetics. Curative treatment generally involves surgery, various forms of radiation therapy, or, less commonly, cryosurgery. Hormonal therapy and chemotherapy are not usually implemented, unless the disease reaches advanced stages and there have been instances where hormonal therapy has been combined with radiation therapy [3].

Over the years, many markers have been used for the diagnosis and follow-up of prostate cancer. Prostate-specific antigen (PSA) is the most common marker used for prostate cancer detection and follow-up, and until recently, PSA was considered the most reliable marker to predict prostate cancer [4]. In 1994, the FDA approved the use of the PSA test in conjunction with a digital rectal exam (DRE) to test asymptomatic men for prostate cancer. Blood PSA levels higher than 4.0 ng/mL is an indication of prostate cancer. Studies have shown that the levels of free PSA in the serum act as a more accurate marker for BPH, while the levels of *α*1-antichymotrypsin-PSA complex more accurately predict prostate cancer [5].

Lately, however, PSA screening has fallen under controversy since it is detected in 30–50% of the cases of benign prostate hyperplasia [BPH] and in only 20% of the cases of prostate cancer. Recent evidence suggests that some prostate cancer patients may present PSA levels below 4.0 ng/mL, while PSA levels can be affected by various other factors, such as prostatitis, urinary tract infection, and benign prostate hyperplasia (BPH) [6–8]. Additionally, a variety of drugs (5*α*-reductase inhibitors, that is, finasteride and dutasteride) used to treat BPH reduce PSA in the blood [9].

Out of the men that display elevated PSA levels in the blood, only 25% are associated with prostate cancer. In order to get more accurate readings on the association between PSA levels and prostate cancer, other factors are taken into consideration, such as free versus total PSA, age (PSA increases with age), PSA velocity and doubling time, pro-PSA, and PSA density of the transition zone [6–8, 10]. Velocity refers to the rate of change in a man's PSA level over time, expressed in (ng/mL)/year, while doubling time refers to the period of time in which the concentration of PSA in the blood doubles. Pro-PSA refers to several inactive PSA precursors that have been suggested to more strongly associate with prostate cancer, while PSA density refers to the blood level of PSA divided by the volume of the interior part of the prostate that surrounds the urethra transition zone.

The absence of a reliable marker for prostate cancer diagnosis and follow-up creates the demand for novel, specific, sensitive, and cost effective biological markers. In this review, we are going to focus on novel biological markers for prostate cancer prognosis and patient follow-up and the possibility to be targeted as markers for prostate cancer treatment.

## 2. The Ideal Marker

Only a few markers have managed to withstand the test of time and entered into clinical trials. The main characteristics of an ideal tumor marker are its specificity for a given tumor type and its sensitivity, and it should also provide advance warning before clinical diagnosis. The levels of the marker should accurately depict the progress or regression of the target tumor. A short half-life would allow for frequent serial measurements. Finally, the detection test should be cheap and noninvasive, so as to allow patient screening and also to be acceptable by the majority of the patients. Finally, tumor-associated markers should be able to predict the metastatic onset or, in advanced stages, determine the metastatic spread [11].

## 3. Current Prostate Cancer Markers

The rapid advancements in overall detection techniques have made it possible to identify a large number of new possible biomarkers; however, a recent study on prostate cancer tissue samples has shown that the equivalence between RNA transcripts and protein products ranges only between 48% and 64% [12]. Since proteins are the true functional molecules of the cell, much of the current research has shifted towards the definition of solely protein markers. The most promising prostate cancer markers among others are the prostate-specific membrane antigen (PSMA), prostate stem cell antigen (PSCA), early prostate cancer antigen (EPCA), enhancer of zeste homolog gene 2 (EZH2), and the urokinase plasminogen activator (uPA) [13, 14].

The PSMA is a type II integral membrane glycoprotein, originally identified in 1987 as being significantly overexpressed in the epithelial cells of prostate cancer patients. Since then, it has undergone multiple evaluations with mixed results. The sensitivity and specificity of PSMA in distinguishing prostate adenocarcinoma from any other type of malignancy are 65.9% and 94.5%, respectively. Some believe that it can be utilized to check the progress of the disease posttreatment. It can also take part in the radiologic imaging of prostate cancer and has been studied as a possible target for monoclonal antibodies to combat prostate cancer, due to its overexpression, despite the fact that its function in prostate cancer is still unclear [15–21].

The PSCA is a prostate-specific glycosyl phosphatidylinositol-anchored glycoprotein expressed on the cell surface. Several studies have shown correlation between increased levels of PSCA and prostate cancer presence, stage, progression, and metastases. Moreover, PSCA RNA is detectable in the peripheral blood through the use of real-time PCR (RT-PCR), an aspect that has been implemented in circulating tumor cell (CTC) detection, while the protein product can act as a target for monoclonal antibodies, as it is situated on the tumor cell surface. As a result, it is a very promising biological marker [22, 23].

The EPCA is a prostate cancer-associated nuclear structural protein. A blood test using an EPCA enzyme-linked immunosorbent assay has displayed 92% sensitivity and 94% specificity for prostate cancer, suggesting a possibly immensely useful biomarker [24].

The EZH2 is a member of the polycomb group of proteins, and it is involved in maintaining the transcriptional repressive state of genes over successive cell generations. EZH2 acts mainly as a gene silencer. EZH2 overexpression may promote cancer due to increase in histone methylation which silences the expression of tumor suppressor genes. Its expression is significantly increased in metastatic prostate cancer in comparison to localized prostate cancer and in localized prostate cancer in comparison to benign prostate tissue [25]. Currently, there is no blood test for EZH2, but it could prove to be a useful biological marker to identify patients at risk of metastasis [13].

The uPA axis is involved in various phases of tumor development and so could act as a potential treatment target. Results show that elevated circulating levels of uPA and uPA receptor (uPAR) are connected with prostate cancer stage and bone metastases. Additionally, uPA has been described as a strong predictor of recurrence after radical prostatectomy [26–28].

Transmembrane protease serine 2 (TMPRSS2) is an enzyme that in humans is encoded by the androgen-regulated TMPRSS2 gene. Its function in prostate cancer lies in the overexpression of E26 transformation-specific (ETS) transcription factors, such as ETS-related gene (ERG) and ETS translocation variant 1 (ETV1) through gene fusion [29]. TMPRSS2-ERG fusion gene is frequently present in human prostate cancer (50%) and it is not detected in normal prostate or BPH [30–33]. It has been suggested that ERG overexpression facilitates prostate cancer progression by promoting androgen independence through disruption of androgen-receptor signaling [29]. Noninvasive detection of TMPRSS2-ERG transcripts is possible in urinary sediments through real-time PCR, presenting a 93% specificity for prostate cancer. This technique is usually carried out in combination with and after digital rectal examination (DRE) [34]. Once combined with prostate cancer antigen 3 (PCA3), the sensitivity increases from 62% (PCA3 alone) to 72% (combined) without sacrificing any of the specificity [35, 36]. These facts constitute TMPRSS2-ERG, a powerful diagnostic tool on its own and a viable way to improve the efficiency of other promising biomarkers.

Studies with general cancer markers are also being performed to determine a possible connection with prostate cancer, aiming to provide accuracy in prostate cancer detection when used solely or in combination with one of the prostate cancer specific markers. The most promising general cancer markers for prostate cancer detection are transforming growth factor-*β*1 (TGF-*β*1) and interleukin-6 (IL-6). TGF-*β*1 is involved in cellular proliferation, redifferentiation, angiogenesis, and epithelial to mesenchymal transition (EMT), the process by which epithelial cells lose cell polarity and cell-to-cell adhesion, gaining migratory and invasive properties, and it has been associated with metastasis in prostate cancer models [37–41]. However, the results are inconclusive regarding its correlation to prostate cancer progression [42, 43]. IL-6 is a cytokine with a large number of biological activities, including regulation of immune response. It has been shown to stimulate cell growth in androgen-independent prostate cancer cells but inhibit it in androgen-dependent prostate cancer cells [44, 45]. Recent studies have introduced the idea of the combined use of TGF-*β*1 and IL-6 to improve the chances of accurately predicting lymph node metastases [46, 47].

Studies have shown that E-cadherin loss correlates with prostate tumor progression, establishing E-cadherin as a prognosis factor for clinical disease progression [48]. On the other hand, the elevation of N-cadherin has been shown to be a significant predictor of prostate cancer recurrence following radical prostatectomy, making it one of the few biomarkers capable of providing information for prostate cancer treatment follow-up [40, 49]. Additional data has shown significant correlation between elevated ZEB1 expression, induced by androgens, and high Gleason scores in prostate cancer [50]. This means that ZEB1 could function as a possible biomarker for predicting the onset of metastatic spread in prostate cancer.

The cancer cells subjected to EMT develop stem-cell-like qualities, practically becoming circulating stem cells. These cells exhibit both tumor and mesenchymal markers [51]. The existence of malignant cells of epithelial origin in the blood, the CTCs, has been known for over a century and has been associated with metastasis. Circulating tumor cell (CTC) counts in the blood have been suggested to act as prostate cancer prognostic markers, especially in cases with bone metastases [52–55]. Over the past few years, different approaches have been developed prior to the detection of CTCs in different tumors. Each of these approaches has distinct advantages and disadvantages, with the most notable being sensitivity and specificity [21, 51, 56–58]. At the moment, there are diagnostic platforms designed to detect CTCs in order to ascertain, up to a point, whether chemotherapy was successful and if there is going to be a cancer recurrence [52, 56].

## 4. The IGF System

The insulin-like growth factor (IGF)/insulin family of growth factors is a system which plays a critical role in the development and growth of several tissues as well as the overall metabolism. It is comprised of three different receptors: the IGF-1 receptor (IGF-1R), IGF-2 receptor (IGF-2R), and the insulin receptor (IR), three different ligands (IGF-1, IGF-2, and insulin), and six types of circulating IGF-binding proteins (IGFBP1-6) [59, 60].

So far, the scientific community is convinced, without data to the contrary, that the IGF-1 system is not, by its nature, oncogenic. The activated receptors are not genotoxic nor do they cause DNA mutations or any other kind of DNA damage [61]. However, they do severely affect the progress of the cell cycle, pushing cells to proliferate at an alarming rate, once their regulation is influenced, like in cases of cancer.

There have been attempts in the past to ascertain whether any part of the IGF axis (ligands, receptors, or binding proteins) could be used as a reliable biological marker for prostate cancer and prostate cancer metastases with controversial results [62, 63]. Since elevated IGF-1 and IGF-1R levels have been associated with many types of cancer and metastases, they cannot be used as prostate cancer markers, at least individually, due to their lack of specificity [59, 60, 63–65]. Certain data showed that the PSA/IGF-1 ratio could differentiate between prostate cancer and BPH but was met with criticism [66]. So far, IGF-1, IGF-1R, and IGFBP3 levels have only been shown to be possible but deficient prostate cancer risk markers. However, there is data that supports the idea that IGF-1 and IGF-1R could be used as biomarkers for advanced stages of prostate cancer and prostate cancer metastases [63, 65, 67]. This could be significant, as compared to some of the other possible biomarkers mentioned.

The phosphorylation of the receptor through the binding of the ligands leads indirectly to the activation of the MAPK/ERK, AKT, and RAS/RAF pathways. This makes the IGF-1R an ideal target for several experimental treatments [59, 60, 68]. Anticancer strategies focusing on the IGF1 signaling system usually belong in one of two categories: neutralizing antibodies and small molecule inhibitors of the IGF-1R kinase activity. Some of them are now being tested at a clinical level, in tandem with standard chemotherapeutic or targeted agents in cancer patients.

Monoclonal antibodies targeting IGF-1R usually target its extracellular domain. Binding of these antibodies has the added effect of downregulating IGF-1R by promoting its internalization. Most antibodies that have been tested in clinical trials have shown no adverse reactions [69]. It was not known until recently that although these antibodies inhibit the binding of the IGF-1 to the IGF-1R, they also activate the IGF-1R (to a lesser extent) by binding to it [70, 71]. A solution to that suggests the use of these antibodies in combination with other antibodies or therapeutic factors targeting the IGF-1R intracellular pathways.

However, IGF-1R is not the only part of the IGF1 axis that has been targeted by neutralizing antibodies. There have been attempts in the past to construct anti-IGF-1 monoclonal antibodies with little success [72, 73]. Nowadays, the focus has shifted entirely towards the IGF-1R.

Along with advancements in analytical technology comes the progress in the characterization of IGF-1R structure [74]. This knowledge facilitates the design and use of small molecule inhibitors targeting IGF-1R. However, it is vitally important that there is no cross-reactivity between them and IR. At the moment, most of these small molecule inhibitors either display high levels of toxicity or they have not made it past stage II clinical trials [67, 75–78].

The anticancer strategies focusing on the IGF-1 system are still in the early stages of research, but their effects on prostate cancer were not associated with a spectacular success. The absence of an alternative, better than PSA, prostate cancer marker, leads to the consideration of other venues of research.

## 5. A Glimpse at the Future

The ideal prostate cancer marker has not been discovered yet. Sometimes, however, just one marker is not enough. This fact gave rise to the idea that the use of multiple markers at the same time could provide improved results. Tumor-associated antigens stimulate the production of autoantibodies (antibodies targeting an individual's own proteins) against cancer [79–82]. The measurement of different anti-tumor autoantibodies, through the use of protein microarrays, is expected to give us autoantibody signatures, that could prove to be a very accurate analytical tool for prostate cancer diagnosis, prognosis, and patient follow-up [83, 84].

Another promising approach towards the discovery of markers, more specific and sensitive than PSA, is the large-scale analysis of prostate cancer proteins, regarding their structures and functions, by proteomics [85]. Several biological sources, including tissues, urine, serum, plasma, and prostatic fluids, are currently under investigation using high-throughput proteomic platforms, such as nanoparticle capture based analysis, for that exact purpose [86]. Secretomics, a subfield of proteomics that studies secreted proteins and secretion pathways using proteomic approaches, has recently emerged as an important tool for the discovery of biomarkers of disease [87].

The prostate has been known for a long time to display unique metabolic profiles [88, 89]. Metabolomics is the study of chemical processes involving metabolites. It is the study of the unique chemical fingerprint that a specific cellular process leaves behind. More specifically, the prostate is unique among human organs due to the high levels of citrate in the prostatic fluid levels that can be 200–700 times higher than the ones in the blood plasma. However, when the prostate is subjected to neoplastic transformation, the prostate's reserves of citrate are depleted due to the increased energy consumption by the rapidly proliferating cancer cells [90, 91].

Quite recently, certain results showed not only that sarcosine, also known as N-methylglycine, an intermediate and byproduct in glycine synthesis and degradation, could be used as a dynamic new biomarker for prostate cancer metastasis, but also that sarcosine levels could control the invasiveness of the cancer. Since then, these results have been widely disputed, while there is doubt that sarcosine is actually an appropriate prostate cancer marker [92–95].

Another marker related to prostate cancer that has surfaced from the realm of metabolomics is choline, a water-soluble essential nutrient. Studies have shown that prostate cancer tissue displays elevated levels of choline and its component metabolites (free choline, phosphocholine, and glycerophosphocholine), in comparison with healthy prostate tissue. These changes reflect enhanced synthesis and degradation of phospholipid membranes. Additionally, levels of choline-containing metabolites are higher in metastatic tissues, when compared to the primary prostate cancer [89, 96–99], indicating the possible use of choline as a prostate cancer progression marker.

Recently, the field of epigenetic modifications has proven to be of interest when it comes to prostate cancer, as they have been connected with both disease initiation and progression [100, 101]. More specifically, DNA-methylation, histone modifications, and microRNA (miRNA) alterations occur at a much higher frequency than mutations and are present at premalignant stages of the disease, making them promising biomarkers [102].

Currently, the most extensively studied methylation-based markers in prostate cancer are the hypermethylated glutathione S-transferase P1 (GSTP1) and Ras-association domain family protein isoform A (RASSF1A). GSTP1 is involved in the cellular protection system against toxic effects and is especially promising as a biomarker because it is highly specific for prostate cancer (>90%); levels of GSTP1 methylation are associated with different stages of the disease; levels of GSTP1 promoter region methylation can differentiate between prostate cancer and BPH and they are detectable by noninvasive means in body fluids [103–106]. The methylation of RASSF1A, on the other hand, can potentially be used to distinguish aggressive tumors from indolent ones [107].

Histone modifications have not been researched to the same extent as methylation-based markers, mostly due to the absence of highly sensitive detection methods [108]. Currently, immunohistochemistry is the only method available for the study of histone modifications, with ELISA being an as of yet unproven alternative [109]. So far, the levels of specific histone modifications, such as H3K18Ac, H4K12Ac, H3K4Me2, and H4R3Me2, have been shown to correlate with prostate cancer tumor stage [110]; but without a reliable method to detect these modifications in biological fluids, progress has been slow. It is clear that this aspect of epigenetic modifications requires further research.

miRNA is also another promising candidate for prostate cancer prognosis and therapy. The mature miRNAs are short, noncoding, single-stranded RNA molecules that bind to complementary sequences in the 3′ UTR of target mRNAs, usually resulting in their silencing. They are detectable in body fluids, such as blood and serum, highly stable due to their placement within microvesicles, and thought to be, in most cases, tumor specific [111, 112]. While a large number of miRNAs have been shown to be altered in prostate cancer, the ones that have displayed the most promise are miR-141 and miR-375 [36]. Further studies have shown that increased expression of miR-141 and miR-375 is significantly associated with pathological stage and Gleason score [113]. Elevated plasma levels of miR-141 and miR-375 could potentially differentiate patients with metastases from those without [114]. Despite the promising results, miRNA implementation in prostate cancer detection is still in its infancy, mainly due to the difficulties in isolating miRNA from limited biological sources.

However, in our quest of discovering and defining new biomarkers, one must take into account the fact that every individual patient is different than the next. Tumors, most commonly, tend to be comprised of multiple cellular clones and this fact may alter the marker expression. There are several lines of evidence in the literature suggesting that the patients' genetic profile could affect patients' response to treatments [64, 115–120]. Therefore it can be understood that identifying new biological markers is clearly not enough and a point of vital importance is to understand how different genetic alterations can influence cancer, so that the most effective course of treatment can be applied.

## 6. Discussion

Despite the fact that NCI does not have such guidelines that suggest the use of markers in cancer, the American Society of Oncology and the National Academy of Clinical Biochemistry have published clinical practice guidelines for markers on a variety of tumors. There are more than 20 tumor markers currently in use, and only the PSA is used in prostate cancer. For the past few years, PSA has raised quite a cloud when it comes to its effectiveness as a biological marker for the detection of prostate cancer. Its deficiencies have given rise to serious efforts to either improve its specificity by combining it with other existing biomarkers or discover and define new ones and also examine the possibility to use those markers as targets for a therapy influencing the balance between benefits (saved lives) and costs (unnecessary surgery).

There are a number of promising markers displayed here that can be used solely or in combination prior to obtaining the desirable result. Despite that, a recent study where 380 prostate cancer markers from the literature were examined in prostate cancer tissues by microarray analysis indicates that none of the markers examined can compete with PSA for tissue specificity. The markers proposed generally presented great variability of expression in normal and tumor tissue or they were expressed at similar levels in other tissues. Furthermore the evidence of this study suggests that the diagnostic and prognostic testing is more difficult in prostate cancer than in other neoplasms probably due to the fact that the individual genetic variability affects the tumor's outcome [121].

For that reason the research for better markers for prostate cancer has been turned towards different markers such as the autoantibodies raised against some tumor markers and/or different technologies proteomics and metabolomics.

Indeed, many of these markers are still in the realm of possibility; but if we take into account the fact that prostate cancer is globally the sixth cancer-associated cause of death in men, its early detection or proper stratification could really make a difference in the socioeconomic system. Therefore, it is imperative that the proper steps are taken to determine which of these markers, if any, would better suit our needs.
